# Cerebral blood flow changes in remitted early- and late-onset depression patients

**DOI:** 10.18632/oncotarget.19185

**Published:** 2017-07-12

**Authors:** Wenxiang Liao, Ze Wang, Xiangrong Zhang, Hao Shu, Zan Wang, Duan Liu, Zhijun Zhang

**Affiliations:** ^1^ Neurologic Department of Affiliated ZhongDa Hospital, Neuropsychiatric Institute and Medical School of Southeast University, Nanjing, Jiangsu 210009, China; ^2^ Center for Cognition and Brain Disorders, Hangzhou Normal University, Affiliated Hospital of Hangzhou Normal University, Hangzhou, Zhejiang 311121, China; ^3^ Department of Geriatric Psychiatry, Affiliated Nanjing Brain Hospital, Nanjing Medical University, Nanjing, Jiangsu 210029, China

**Keywords:** early-onset depression, late-onset depression, cerebral blood flow

## Abstract

Abnormal cerebral blood flow (CBF) is reportedly associated with major depressive disorder (MDD). We have investigated CBF changes in early-onset depression (EOD) and late-onset depression (LOD), and their impact on cognitive function. Thirty-two remitted EOD patients, 32 remitted LOD patients, and 43 age-matched healthy controls were recruited, and the pulsed arterial spin labeling data were scanned under 3.0T MRI and processed through voxel-by-voxel statistical analysis. Compared to healthy controls, LOD patients had decreased normalized CBF in the bilateral precuneus, cuneus, right fronto-cingulate-striatal areas, and right temporal, occipital and parietal lobes, but increased normalized CBF in the left frontal and temporal cortices and the cingulate gyrus. EOD patients had decreased normalized CBF in the left cerebellum and right calcarine/lingual/fusiform gyrus, and increased normalized CBF in right angular gyrus. LOD patients displayed hemispheric asymmetry in CBF, and had more regions with abnormal CBF than EOD patients. A significant correlation between abnormal CBF and impaired cognitive function was detected in LOD patients, but not EOD patients. These results demonstrate greater CBF abnormalities in LOD patients than EOD patients, and suggest these CBF changes may be associated with progressive degradation of cognitive function in LOD patients.

## INTRODUCTION

Late life depression (LLD) is a common mood disorder with a pervasive and persistent low mood in elderly people (60 years or older). It is invariably accompanied by multiple neuropsychology impairments and abnormal neuroimaging results [1–3]. According to the age at depression onset, LLD could be divided into late onset (first onset at age 60 or above) depression (LOD) and early onset (first onset before age 60) depression (EOD). Previous studies have reported an association between cerebrovascular disease and late life depression [4, 5]. Relative to EOD patients, LOD patients have shown more vascular changes [6, 7], greater cognitive impairments [8, 9] hippocampal atrophy [10, 11], and higher risk of dementia [12]. In addition, cerebrovascular changes contribute to the development of depressive symptoms in patients with late life depression [13].

Cerebral blood flow (CBF) reflects the neural activity through the neurovascular coupling and has traditionally been measured using nuclear medicine techniques, such as single photon emission computed tomography (SPECT) and positron emission tomography (PET). Significant reductions of regional CBF in the frontal, parietal, and temporal regions have been found in major depressive disorder (MDD) patients [14, 15]. Unlike PET and SPECT techniques, arterial spin labeling (ASL) magnetic resonance imaging (MRI) offers a novel noninvasive approach to quantitatively measure CBF [16, 17]. The results of ASL have comparable diagnostic accuracy with PET [18–20]. Recently, ASL has been applied to investigate brain function in MDD patients, but the CBF data have varied considerably. Several studies have reported decreased CBF perfusion in the frontal regions and anterior cingulate cortex (ACC) in MDD patients [21–24], while other studies have reported opposite results [25]. Both increased [23] and decreased [21] CBF perfusion has been reported in limbic areas in MDD patients. ASL analysis of CBF perfusion in LLD patients has been rarely used, and more studies are needed [26]. Specifically, it is important to analyze the relationship between CBF and cognitive function in LLD patients, especially in the two subgroups, LOD and EOD patients.

Cerebral blood flow varies substantially between acute and remitted MDD patients. A previous study reported that abnormal CBF of most brain regions including frontal, temporal and cingulate cortices was improved, while decreased CBF was persistent in other regions, such as right lingual gyrus following 6-week antidepressant treatment [27]. An increased CBF in the dorsolateral prefrontal cortex was found in MDD adolescents after a five-session cognitive behavioral group therapy, which coincided with a reduction in MDD symptoms [28]. Our group has recently reported that the deficit in executive function and memory persisted in the remitted late life depression, which might be implicated in gradual conversion to dementia [29]. Therefore, it is important to elucidate the CBF abnormalities and their potential influence on permanent cognitive impairment in remitted elderly MDD patients. In this study, we used pulsed-ASL (pASL) to compare the grey matter CBF levels between remitted LOD, EOD, and age-matched healthy controls (HC). We hypothesized that there were different regional CBF levels, possibly involving fronto-limbic-cingulate-striatal areas among the three groups. Furthermore, we explored the correlation between abnormal regional perfusion and cognitive dysfunction in the remitted LOD and EOD patients.

## RESULTS

### Demographic data and neuropsychological performance in LOD, EOD, and HC groups

There were no significant differences in gender, age, years of education, and the results of the TMT-A test (in seconds) among the LOD, EOD, and HC groups. However, both LOD and EOD patients performed significantly worse on Hamilton depression rating scale (HAMD), auditory verbal learning test-delay recall (AVLT-DR), Rey-Osterrieth complex figure test-delay recall (ROCF-DR), and semantic similarity test when compared with healthy controls. In addition, LOD patients showed poorer performance on digit symbol substitution test (DSST) and TMT-B test (in seconds) than healthy controls. However, no significant differences in cognitive performance were found between LOD and EOD groups (Table 1).

**Table 1:** Demographic and neuropsychological data among LOD, EOD and HC groups

	LOD (*n* = 32)	EOD (*n* = 32)	HC (*n* = 43)	χ^2^/F/t	*p*
Sex(male/female)	10/22	8/24	16/27	1.3	0.531^a^
Age	69.1 ± 5.3	66.1 ± 5.6	67.8 ± 5.3	2.6	0.079^b^
Education	11.6 ± 2.9	11.6 ± 2.6	12.1 ± 2.4	0.5	0.620^b^
HAMD	3.8 ± 4.0*	4.2 ± 2.3^#^	1.5 ± 2.4	9.0	< 0.001^b^
Age at onset	67.7 ± 5.2	39.6 ± 8.4	-	16.1	< 0.001^c^
Duration of depression	1.5 ± 1.1	26.5 ± 11.4	-	–12.3	< 0.001^c^
AVLT-DR	6.0 ± 2.3^**^	5.8 ± 2.9^##^	8.0 ± 1.6	10.9	< 0.001^b^
DSST	31.5 ± 9.3^**^	36.4 ± 10.3	41.1 ± 9.0	9.4	< 0.001^b^
ROCF-DR	13.3 ± 6.1^**^	12.5 ± 7.5^##^	19.8 ± 4.9	16.4	< 0.001^b^
Semantic similarity	16.2 ± 4.8^**^	16.9 ± 2.3^##^	20.1 ± 2.5	15.4	< 0.001^b^
TMT-A (seconds)	74.5 ± 27.5	67.8 ± 16.8	65.1 ± 16.0	2.0	0.138^b^
TMT-B (seconds)	207.3 ± 73.7*	185.9 ± 67.5	166.2 ± 55.0	3.7	0.028^b^

Note: LOD: late-onset depression; EOD: early-onset depression; HC: healthy controls; HAMD: Hamilton depression scale; AVLT-DR: auditory verbal learning test-delay recall; DSST: digit symbol substitution test; ROCF-DR: Rey-Osterrieth complex figure fest-delay recall; TMT: trail making test. Post hoc analysis: LOD vs HC: *p < 0.05; ^**^p < 0.001; EOD vs HC: ^#^*p <* 0.05; ^##^*p <* 0.001. ^a^Chi square test; ^b^Analysis of variance; ^c^Independent samples *t*-test.

### Normalized CBF values in LOD, EOD, and HC groups

**Figure 1:**
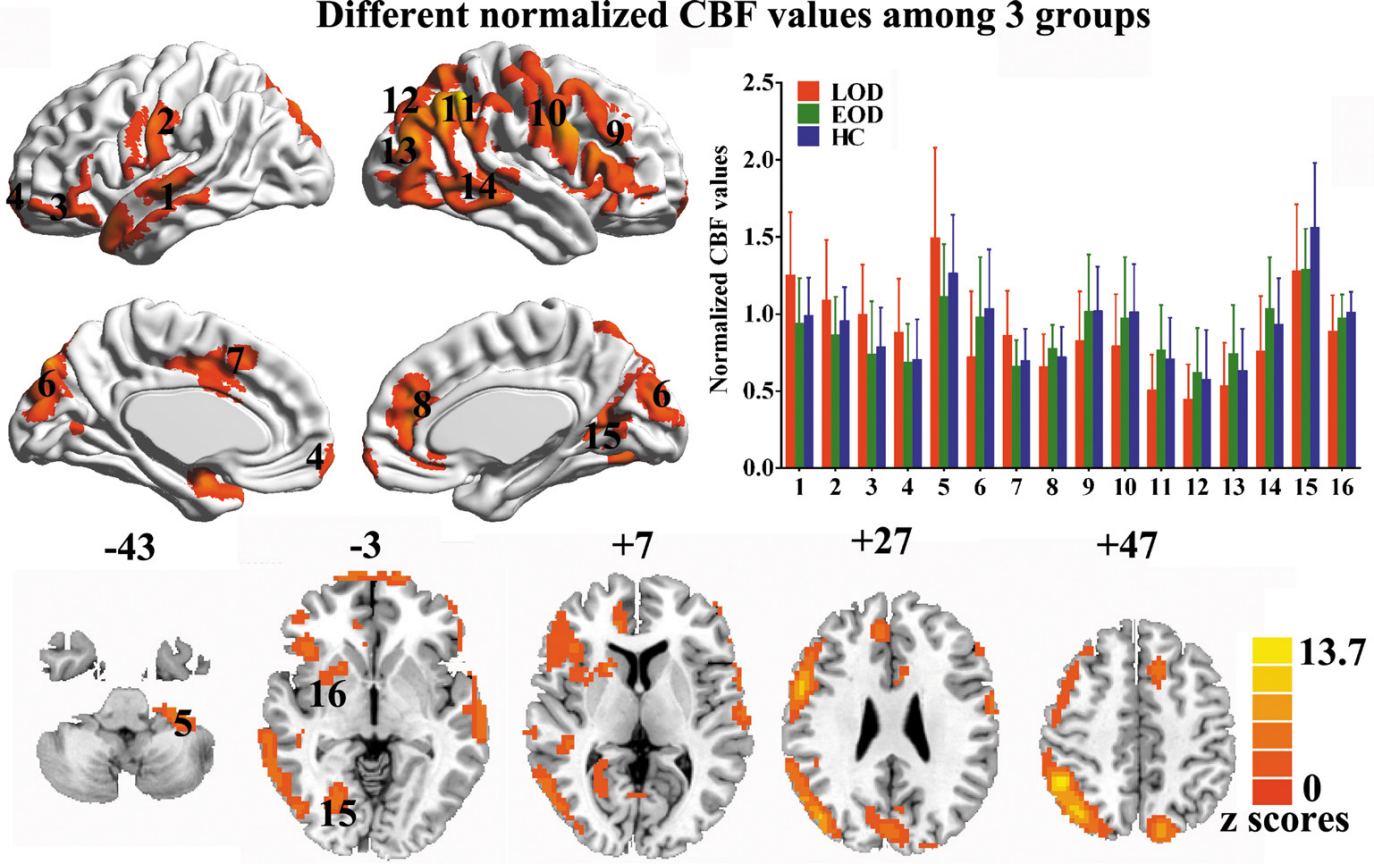
Maps of normalized CBF analyzed by ANCOVA with gender, age, and education as covariates, in LOD, EOD, and HC groups Data were corrected by Monte Carlo simulation with *p <* 0.05 and a minimum cluster of 3807 mm^3^. (1 = left lateral temporal cortex, 2 = left precentral/postcentral gyrus, 3 = left lateral orbitofrontal cortex, 4 = medial orbitofrontal cortex, 5 = left cerebellum, 6 = bilateral cuneus, 7 = middle cingulate cortex/supplementary motor area, 8 = anterior cingulate cortex, 9 = right frontal cortex, 10 = right precentral/postcentral gyrus, 11 = right parietal lobe, 12 = bilateral precuneus, 13 = right lateral occipital cortex, 14 = right lateral temporal cortex, 15 = right calcarine/lingual/fusiform gyrus, 16 = right insula/striatum.)

**Figure 2:**
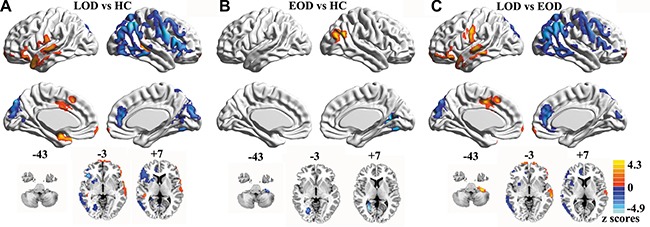
Maps of normalized CBF analyzed by post-hoc analysis in LOD, EOD, and HC groups Data were corrected by Monte Carlo simulation with *p <* 0.05 and a minimum cluster size of 1269 mm^3^.

The normalized CBF values differed in cerebral cortices, including frontal, temporal, parietal, occipital, cingulate cortices, and insula/striatum (Figure 1). The post hoc analysis showed that LOD patients had significantly increased CBF in left frontal, temporal, cingulate cortices and right superior temporal gyrus, and decreased CBF in bilateral precuneus, cuneus and right cerebral cortices when compared with healthy controls (Figure 2A). Similarly, EOD patients had increased CBF in right angular gyrus and decreased CBF in left cerebellum and right medial occipital cortex relative to healthy controls (Figure 2B). Compared with EOD patients, LOD patients had increased normalized CBF values in left frontal, temporal, cingulate cortices, and cerebellum, while decreased normalized CBF in bilateral precuneus, cuneus, and right cerebral cortices (Figure 2C).

### Partial correlation analysis in LOD, EOD, and HC groups

**Figure 3:**
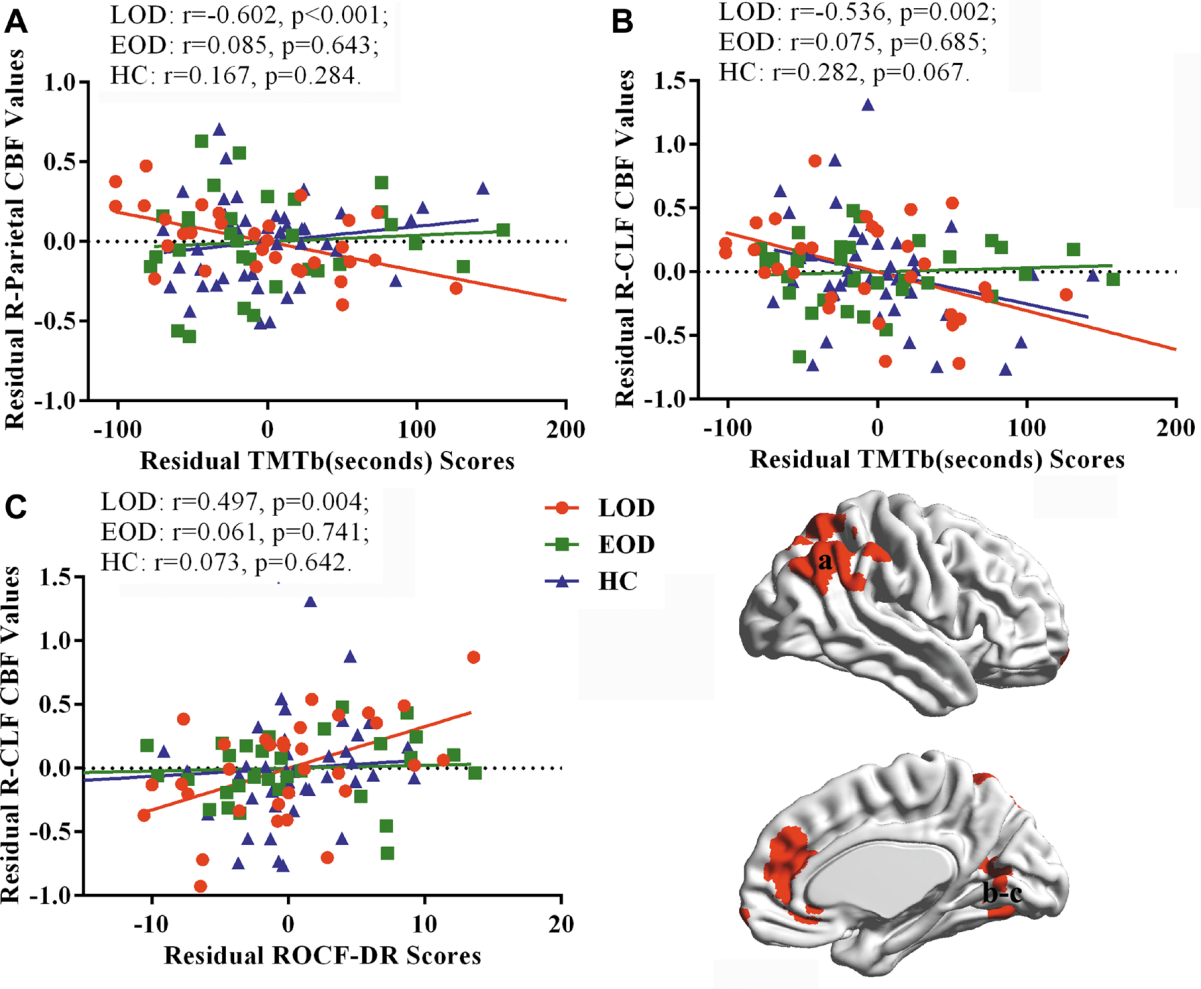
Results of partial correlation analysis with gender, age, and education as covariates (TMT-B: trail making test-part B, R-CLF: right calcarine/lingual/fusiform gyrus, ROCF-DR: Rey-Osterrieth complex figure test-delay recall)

The partial correlation analysis revealed that the TMT-B test scores negatively correlated with the normalized CBF values in right parietal cortex and calcarine/lingual/fusiform gyrus (CLF) in LOD patients. Additionally, normalized CBF values in right CLF positively correlated with ROCF-DR scores in LOD patients. These brain-behavior correlations were not observed in EOD patients and healthy controls (Figure 3).

## DISCUSSION

Using ASL imaging technique we found significant CBF changes in several brain regions of patients with remitted LOD and EOD compared with healthy control (HC) individuals. Remarkably, LOD patients had increased CBF in left brain areas, but decreased CBF in right brain areas. More differences in CBF were found between LOD and EOD patients. To our knowledge, this is the first report demonstrating different characteristics of normalized CBF between remitted LOD and EOD patients. In addition, abnormalities in normalized CBF correlated with cognitive impairment in LOD patients, but not in EOD patients.

We have found decreased normalized CBF of right calcarine/lingual/fusiform gyrus both in remitted LOD and EOD patients. Consistently, decreased CBF in right lingual gyrus has been reported in patients with acute depression, which persisted after 6-week antidepressant treatment [27]. The decreased CBF of right lingual gyrus might be a common characteristic of brain hemodynamics regardless of the two subtypes of depression. However, this study has demonstrated inconsistent changes in CBF in several brain regions between EOD and LOD patients. First, the CBF in right angular gyrus was increased in EOD patients, but decreased in LOD patients. It has been previously reported that the right angular gyrus exhibits a decreased regional spontaneous neural activity (ReHo) in treatment-naive LOD patients [30], while it has increased ReHo in young adults with MDD [31]. Second, the present study revealed the reduction of CBF mainly in the right fronto-cingulate-limbic-striatal areas in remitted LOD but not EOD patients. A previous study has reported decreased CBF in frontal and anterior cingulate cortices in acute phase of late life depression [32]. Compared with EOD, LOD has a higher level of comorbidity, such as cerebrovascular disease and carotid plaques [6, 7]. Vascular depression has been associated with older age, late age at onset, and vascular risk factors (e.g., white matter hyperintensities, cerebrovascular disease, diabetes, and hypertension) [5, 33, 34]. The present results support the vascular depression hypothesis [33], with the disruption of prefrontal systems or their modulating pathways in LOD. Studies focusing on CBF in remitted LOD patients are virtually absent. Kaichi et al. [27] have reported that abnormal CBF improved in most brain areas including frontal, temporal cortices, and ACC, after 6-week antidepressant treatment in youth patients with MDD. These results, along with our findings, suggest an improved CBF in the remitted EOD patients.

A significant CBF perfusion asymmetry was found in remitted LOD patients who had increased CBF in left brain areas, but decreased CBF in right frontal and temporal cortices. In this regard, hemispheric asymmetry has been previously reported in MDD patients. Consistently with our findings, Kaichi et al. [27] have found an increased CBF in left hemisphere and decreased CBF in right hemisphere in MDD patients. A reduced excitability of both excitatory (rest motor threshold and intra-cortical facilitation) and inhibitory (corticospinal silent period and intra-cortical inhibition) processes in left hemisphere compared with right hemisphere was reported in MDD patients [35]. MDD patients exhibited hemispheric lateralization of emotion processing; right, but not left hemisphere, was preferentially sensitive to affective context [36]. However, a recent meta-analysis reported an opposite CBF perfusion asymmetry pattern in medication-free patients with MDD, showing increased CBF in right thalamus, caudate and precuneus, and decreased CBF in left middle frontal gyrus, insula and superior temporal gyrus [37]. This meta-analysis included studies focusing on the acute phase of young adult patients with depression, suggesting that CBF perfusion might be affected by the onset age and the therapeutic status of depressive symptoms. Therefore, hemispheric asymmetry may be an important characteristic in remitted LOD patients.

The present study found that the remitted LOD patients had worse executive function (TM*T*-test B scores) and memory (ROCF-DR scores), while the remitted EOD patients had decreased ROCF-DR scores relative to healthy controls. Consistently, executive function and memory impairments have been reported to be persistent in remitted depression [38, 39]. LOD patients had more serious cognitive impairment [8, 9] and higher risk of dementia than EOD patients [12]. Furthermore, our study has demonstrated that abnormal normalized CBF correlates with TM*T*-test B and ROCF-DR scores in remitted LOD, but not EOD patients. The inter-network connectivity has been previously found to correlate with executive dysfunction in late life depression [40], and the abnormal global topology of default mode network correlates with memory deficit in remitted LOD [41]. Consistent with our results, a previous study has found that cognitive function correlates with brain structure alterations (including prefrontal cortical and hippocampal) in LOD, but not EOD patients [42]. The ASL technique may be preferentially sensitive to the earliest pathological events of cognitive disorder prior to macroscopic grey matter loss [43]. Our findings indicate that the robust CBF disturbance might be implicated in the cognitive decline in LOD patients.

However, there are several limitations in the current study. First, due to a cross-sectional study design, we could not determine whether the observed CBF changes in patients with remitted LOD and EOD reflect the state or the trait of the disease. The correlation between CBF and cognitive function in remitted LOD patients might suggest a possibility of regional perfusion reduction together with progressive cognitive impairments across time; this should be addressed in future research. Second, previous work has reported that antidepressants may induce significant reduction of CBF in several brain regions including the amygdala, fusiform gyrus, insula, and orbitofrontal cortices in healthy subjects [44]. Since most recruited patients received antidepressants, it is conceivable that the influence of these medications might have contributed to the alterations of regional blood perfusion. Third, the present study used pASL sequence instead of pseudo-continuous ASL (pCASL) sequence. The pCASL sequence has higher signal-to-noise ratio (SNR) than the pASL sequence [45]. Future research should use pCASL sequence to investigate the CBF alterations. Additionally, in the present study, the labeling parameters (TI1:600 ms; TI2: 1600 ms) were shorter than previous recommendations (TI1:800 ms; TI2: 2000 ms in adult clinical patients) [46]. When TI2 is shorter than arterial transit time (ATT), low ASL signal may reflect the combination of low CBF and unexpectedly long ATT. ATT varies between 500–1500 ms in healthy gray matter, but can be 2000 ms or longer in cerebrovascular disease and in deep white matter. Therefore, some areas with decreased CBF might have resulted from inadequate TI2.

In conclusion, this study has detected increased abnormalities and hemispheric asymmetry in CBF in remitted LOD patients compared to EOD patients. LOD patients exhibited a correlation between CBF alterations and cognitive impairment. Further studies are needed to investigate the molecular mechanisms responsible for the CBF differences between LOD and EOD patients, and analyze the relationship between neuroimaging characteristics and cognitive functions in LOD patients.

## MATERIALS AND METHODS

### Participants

The study included 107 naturally right-handed Han Chinese participants, including 64 patients with remitted late life depression who were recruited from the geriatric psychiatry outpatient centers at Affiliated ZhongDa Hospital, Southeast University and Affiliated Brain Hospital, Nanjing Medical University, and 43 non-depressed healthy controls (HC) recruited through local advertisements. The remitted LLD patients had a history of depressive episode, which met the diagnosis of unipolar major depressive disorder based on the Diagnostic and Statistical Manual of Mental Disorders, Fourth Edition, Text Revision (DSM-IV-TR). The remitted status was diagnosed when the LLD patients had Hamilton depression rating scale for depression-17 items (HAMD-17) scores ≤ 7 for more than 2 consecutive months. The remitted LLD patients were divided into remitted EOD patients (*n* = 32) and remitted LOD patients (*n* = 32) based on the age of first onset. All participants met the following criteria: (1) age between 60 and 80; (2) at least 8 years of education; (3) HAMD scores less than or equal to 7; (4) absence of dementia. The exclusion criteria were as follows: (1) past or current history of any other DSM-IV-TR Axis I psychiatric disorders, such as psychotic or bipolar disorder, alcohol or substance abuse/dependence; (2) history of neurological disease including Parkinson's disease, multiple sclerosis, epilepsy, or stroke; (4) head injury with loss of consciousness; (5) severe visual or hearing loss; (6) MRI contraindications. All participants provided written informed consent and the study protocol was approved by the Hospital Ethics Committee in accordance with the Declaration of Helsinki.

### Neuropsychological assessments

Neuropsychological assessments of all participants were carried out by a highly trained examiner to evaluate the function of episodic memory, executive function, and processing speed. The neuropsychological measures consisted of auditory verbal learning test-delay recall (AVLT-DR), digit symbol substitution test (DSST), Rey-Osterrieth complex figure test-delay recall (ROCF-DR), semantic similarity, and trail making test - parts A and B (TMT-A,B). Table 1 contains demographic and neuropsychological data for all participants.

### MRI data acquisition

All participants underwent an MRI scan, which was performed on a 3.0 Tesla Trio Siemens scanner with a 12-channel head-coil (Siemens, USA) at Affiliated ZhongDa Hospital, Southeast University. Pulsed arterial spin labeled (pASL) images were acquired using a PICORE Q2T sequence (TR = 4000 ms, TE = 12 ms, TI1 = 600 ms, TI2 = 1600 ms, number of slices = 27, thickness = 4 mm, gap = 1 mm, imaging matrix = 64 × 64, FOV = 250 mm × 250 mm, FA = 90°, acquisition duratio*n* = 7 min and 14s). High-resolution T1-weighted axial images covering the whole brain were acquired using a T1-weighted 3D-MPRAGE sequence (TR = 1900 ms, TE = 2.48 ms, TI = 900 ms, flip angle (FA) = 9°, number of slices = 176, thickness = 1mm, gap = 0mm, imaging matrix = 256 × 256, field of view (FOV) = 250 mm × 250 mm, acquisition duration: 4 min and 18 s).

### MRI data preprocessing

The ASL data preprocessing was carried out using ASL toolbox [47], which is based on SPM8 (http://www.fil.ion.ucl.ac.uk/spm). The first volume of 105 ASL acquisitions was used as the M0 image with the remaining 104 volumes used as 52 control-label pairs. A six-parameter rigid body motion spatial transformation was used in aligning the raw echo-planar imaging (EPI) time series. The spurious motion component caused by the systematic label/control alternation was regressed out from the motion parameters before applying the transformation on the images [48]. The next processing consisted of co-registering the mean EPI images to the high-resolution T1 images, which were simultaneously segmented into gray matter (GM), white matter (WM), and cerebrospinal fluid tissue probability maps (TPMs). Image masks and the TPMs were resliced to the native ASL spaces.

The M0 images were co-registered to mean EPI images and smoothed with full-width-half-max (FWHM) = 6 mm isotropic Gaussian kernel. Pairwise subtraction of the control-label images was performed and the difference images were converted to absolute CBF maps using the formula [46]

$$\text{CBF }\left(\text{ml/ 100g/ min}\right)\;=\frac{60\;\times \;100\lambda \text{Δ}Me^{{\omega /T}_{1,\;blood}}}{2\alpha \tau \;M_{0}}$$

where λ is the brain-blood partition coefficient, ΔM is control-label difference, ω is the post-labeling delay, T_1,blood_ is the T1 of blood, α is the tagging efficiency, τ is the labeling duration, and M_0_ is the equilibrium magnetization of the brain. In the present study, λ = 0.9 ml/g, ω = 1.6 sec, T_1, blood_ = 1650 msec, α = 0.95, and τ = 600 msec. The CBF maps were normalized by dividing by the global mean CBF value of each participant to reduce the inter-individual variability.

The CBF images were processed using partial volume correction in the native ASL spaces and subsequently normalized to Montreal Neurological Institute (MNI) space using a linear affine transformation. The images in the MNI space were smoothed using an isotropic Gaussian kernel with FWHM = 6 mm.

### Statistical analysis

The package PASW statistics 18.0 software was used to assess the differences in descriptive demographic and neuropsychological data using analysis of variance (ANOVA), independent samples *t*-test, and chi-squared tests among the three groups. Group differences in normalized CBF were investigated using analysis of covariance (ANCOVA) with controlling the covariates of gender, age and education (A single voxel threshold was set at *p <* 0.05 and corrected by Monte Carlo simulation). To investigate the correlation between abnormal perfusion areas and cognitive impairments, partial correlative analysis [49] was used separately with gender, age, and education as covariates in the three groups. Similarly, the partial correlative analysis was performed to investigate the association between clinical variables (age of depression onset and illness duration) and different normalized CBF values in the LOD and EOD groups.

## References

[1] Kohler S, Thomas AJ, Barnett NA, O'Brien JT (2010). The pattern and course of cognitive impairment in late-life depression. Psychol Med.

[2] Herrmann LL, Goodwin GM, Ebmeier KP (2007). The cognitive neuropsychology of depression in the elderly. Psychol Med.

[3] Mackin RS, Nelson JC, Delucchi KL, Raue PJ, Satre DD, Kiosses DN, Alexopoulos GS, Arean PA (2014). Association of age at depression onset with cognitive functioning in individuals with late-life depression and executive dysfunction. Am J Geriatr Psychiatry.

[4] Thomas AJ, Kalaria RN, O'Brien JT (2004). Depression and vascular disease: what is the relationship?. J Affect Disord.

[5] Sheline YI, Pieper CF, Barch DM, Welsh-Bohmer K, McKinstry RC, MacFall JR, D'Angelo G, Garcia KS, Gersing K, Wilkins C, Taylor W, Steffens DC, Krishnan RR (2010). Support for the vascular depression hypothesis in late-life depression: results of a 2-site, prospective, antidepressant treatment trial. Arch Gen Psychiatry.

[6] Laks J, Engelhardt E (2010). Peculiarities of geriatric psychiatry: a focus on aging and depression. CNS Neurosci Ther.

[7] Paranthaman R, Burns AS, Cruickshank JK, Jackson A, Scott ML, Baldwin RC (2012). Age at onset and vascular pathology in late-life depression. Am J Geriatr Psychiatry.

[8] Mackin RS, Nelson JC, Delucchi KL, Raue PJ, Satre DD, Kiosses DN, Alexopoulos GS, Arean PA (2014). Association of Age at Depression Onset with Cognitive Functioning in Individuals with Late-Life Depression and Executive Dysfunction. Am J Geriatr Psychiatry.

[9] Herrmann LL, Goodwin GM, Ebmeier KP (2007). The cognitive neuropsychology of depression in the elderly. Psychol Med.

[10] Lloyd AJ, Ferrier IN, Barber R, Gholkar A, Young AH, O'Brien JT (2004). Hippocampal volume change in depression: late- and early-onset illness compared. Br J Psychiatry.

[11] Sachs-Ericsson N, Corsentino E, Moxley J, Hames JL, Rushing NC, Sawyer K, Joiner T, Selby EA, Zarit S, Gotlib IH, Steffens DC (2013). A longitudinal study of differences in late- and early-onset geriatric depression: depressive symptoms and psychosocial, cognitive, and neurological functioning. Aging Ment Health.

[12] Sachs-Ericsson N, Moxley JH, Corsentino E, Rushing NC, Sheffler J, Selby EA, Gotlib I, Steffens DC (2014). Melancholia in later life: late and early onset differences in presentation, course, and dementia risk. Int J Geriatr Psychiatry.

[13] Salloway S, Malloy P, Kohn R, Gillard E, Duffy J, Rogg J, Tung G, Richardson E, Thomas C, Westlake R (1996). MRI and neuropsychological differences in early- and late-life-onset geriatric depression. Neurology.

[14] Smith DJ, Cavanagh JT (2005). The use of single photon emission computed tomography in depressive disorders. Nucl Med Commun.

[15] Drevets WC, Bogers W, Raichle ME (2002). Functional anatomical correlates of antidepressant drug treatment assessed using PET measures of regional glucose metabolism. Eur Neuropsychopharmacol.

[16] Detre JA, Leigh JS, Williams DS, Koretsky AP (1992). Perfusion imaging. Magn Reson Med.

[17] Williams DS, Detre JA, Leigh JS, Koretsky AP (1992). Magnetic resonance imaging of perfusion using spin inversion of arterial water. Proc Natl Acad Sci USA.

[18] Musiek ES, Chen Y, Korczykowski M, Saboury B, Martinez PM, Reddin JS, Alavi A, Kimberg DY, Wolk DA, Julin P, Newberg AB, Arnold SE, Detre JA (2012). Direct comparison of fluorodeoxyglucose positron emission tomography and arterial spin labeling magnetic resonance imaging in Alzheimer's disease. Alzheimers Dement.

[19] Chen Y, Wolk DA, Reddin JS, Korczykowski M, Martinez PM, Musiek ES, Newberg AB, Julin P, Arnold SE, Greenberg JH, Detre JA (2011). Voxel-level comparison of arterial spin-labeled perfusion MRI and FDG-PET in Alzheimer disease. Neurology.

[20] Zhang K, Herzog H, Mauler J, Filss C, Okell TW, Kops ER, Tellmann L, Fischer T, Brocke B, Sturm W, Coenen HH, Shah NJ (2014). Comparison of cerebral blood flow acquired by simultaneous [15O] water positron emission tomography and arterial spin labeling magnetic resonance imaging. J Cereb Blood Flow Metab.

[21] Ho TC, Wu J, Shin DD, Liu TT, Tapert SF, Yang G, Connolly CG, Frank GK, Max JE, Wolkowitz O, Eisendrath S, Hoeft F, Banerjee D (2013). Altered cerebral perfusion in executive, affective, and motor networks during adolescent depression. J Am Acad Child Adolesc Psychiatry.

[22] Jarnum H, Eskildsen SF, Steffensen EG, Lundbye-Christensen S, Simonsen CW, Thomsen IS, Frund ET, Theberge J, Larsson EM (2011). Longitudinal MRI study of cortical thickness, perfusion, and metabolite levels in major depressive disorder. Acta Psychiatr Scand.

[23] Lui S, Parkes LM, Huang X, Zou K, Chan RC, Yang H, Zou L, Li D, Tang H, Zhang T, Li X, Wei Y, Chen L (2009). Depressive disorders: focally altered cerebral perfusion measured with arterial spin-labeling MR imaging. Radiology.

[24] Ota M, Noda T, Sato N, Hattori K, Teraishi T, Hori H, Nagashima A, Shimoji K, Higuchi T, Kunugi H (2014). Characteristic distributions of regional cerebral blood flow changes in major depressive disorder patients: a pseudo-continuous arterial spin labeling (pCASL) study. J Affect Disord.

[25] Duhameau B, Ferre JC, Jannin P, Gauvrit JY, Verin M, Millet B, Drapier D (2010). Chronic and treatment-resistant depression: a study using arterial spin labeling perfusion MRI at 3Tesla. Psychiatry Res.

[26] Colloby SJ, Firbank MJ, He J, Thomas AJ, Vasudev A, Parry SW, O'Brien JT (2012). Regional cerebral blood flow in late-life depression: arterial spin labelling magnetic resonance study. Br J Psychiatry.

[27] Kaichi Y, Okada G, Takamura M, Toki S, Akiyama Y, Higaki T, Matsubara Y, Okamoto Y, Yamawaki S, Awai K (2016). Changes in the regional cerebral blood flow detected by arterial spin labeling after 6-week escitalopram treatment for major depressive disorder. J Affect Disord.

[28] Sosic-Vasic Z, Abler B, Gron G, Plener P, Straub J (2017). Effects of a brief cognitive behavioural therapy group intervention on baseline brain perfusion in adolescents with major depressive disorder. Neuroreport.

[29] Liao W, Zhang X, Shu H, Wang Z, Liu D, Zhang Z (2017). The characteristic of cognitive dysfunction in remitted late life depression and amnestic mild cognitive impairment. Psychiatry Res.

[30] Liu F, Hu M, Wang S, Guo W, Zhao J, Li J, Xun G, Long Z, Zhang J, Wang Y, Zeng L, Gao Q, Wooderson SC (2012). Abnormal regional spontaneous neural activity in first-episode, treatment-naive patients with late-life depression: a resting-state fMRI study. Prog Neuropsychopharmacol Biol Psychiatry.

[31] Liang MJ, Zhou Q, Yang KR, Yang XL, Fang J, Chen WL, Huang Z (2013). Identify changes of brain regional homogeneity in bipolar disorder and unipolar depression using resting-state FMRI. PLoS One.

[32] Ishizaki J, Yamamoto H, Takahashi T, Takeda M, Yano M, Mimura M (2008). Changes in regional cerebral blood flow following antidepressant treatment in late-life depression. Int J Geriatr Psychiatry.

[33] Alexopoulos GS, Meyers BS, Young RC, Campbell S, Silbersweig D, Charlson M (1997). 'Vascular depression' hypothesis. Arch Gen Psychiatry.

[34] Krishnan KR, Hays JC, Blazer DG (1997). MRI-defined vascular depression. Am J Psychiatry.

[35] Lefaucheur JP, Lucas B, Andraud F, Hogrel JY, Bellivier F, Del CA, Rousseva A, Leboyer M, Paillere-Martinot ML (2008). Inter-hemispheric asymmetry of motor corticospinal excitability in major depression studied by transcranial magnetic stimulation. J Psychiatr Res.

[36] Atchley RA, Ilardi SS, Enloe A (2003). Hemispheric asymmetry in the processing of emotional content in word meanings: the effect of current and past depression. Brain Lang.

[37] Chen ZQ, Du MY, Zhao YJ, Huang XQ, Li J, Lui S, Hu JM, Sun HQ, Liu J, Kemp GJ, Gong QY (2015). Voxel-wise meta-analyses of brain blood flow and local synchrony abnormalities in medication-free patients with major depressive disorder. J Psychiatry Neurosci.

[38] Yeh YC, Tsang HY, Lin PY, Kuo YT, Yen CF, Chen CC, Liu GC, Chen CS (2011). Subtypes of mild cognitive impairment among the elderly with major depressive disorder in remission. Am J Geriatr Psychiatry.

[39] Sexton CE, McDermott L, Kalu UG, Herrmann LL, Bradley KM, Allan CL, Le Masurier M, Mackay CE, Ebmeier KP (2012). Exploring the pattern and neural correlates of neuropsychological impairment in late-life depression. Psychol Med.

[40] Li W, Wang Y, Ward BD, Antuono PG, Li SJ, Goveas JS (2017). Intrinsic inter-network brain dysfunction correlates with symptom dimensions in late-life depression. J Psychiatr Res.

[41] Yin Y, Wang Z, Zhang Z, Yuan Y (2016). Aberrant topographical organization of the default mode network underlying the cognitive impairment of remitted late-onset depression. Neurosci Lett.

[42] Lebedeva A, Borza T, Haberg AK, Idland AV, Dalaker TO, Aarsland D, Selbaek G, Beyer MK (2015). Neuroanatomical correlates of late-life depression and associated cognitive changes. Neurobiol Aging.

[43] Habib M, Mak E, Gabel S, Su L, Williams G, Waldman A, Wells K, Ritchie K, Ritchie C, O'Brien JT (2017). Functional neuroimaging findings in healthy middle-aged adults at risk of Alzheimer's disease. Ageing Res Rev.

[44] Chen Y, Wan HI, O'Reardon JP, Wang DJ, Wang Z, Korczykowski M, Detre JA (2011). Quantification of cerebral blood flow as biomarker of drug effect: arterial spin labeling phMRI after a single dose of oral citalopram. Clin Pharmacol Ther.

[45] Wu WC, Fernandez-Seara M, Detre JA, Wehrli FW, Wang J (2007). A theoretical and experimental investigation of the tagging efficiency of pseudocontinuous arterial spin labeling. Magn Reson Med.

[46] Alsop DC, Detre JA, Golay X, Gunther M, Hendrikse J, Hernandez-Garcia L, Lu H, MacIntosh BJ, Parkes LM, Smits M, van Osch MJ, Wang DJ, Wong EC (2015). Recommended implementation of arterial spin-labeled perfusion MRI for clinical applications: A consensus of the ISMRM perfusion study group and the European consortium for ASL in dementia. Magn Reson Med.

[47] Wang Z, Aguirre GK, Rao H, Wang J, Fernandez-Seara MA, Childress AR, Detre JA (2008). Empirical optimization of ASL data analysis using an ASL data processing toolbox: ASLtbx. Magn Reson Imaging.

[48] Wang Z (2012). Improving cerebral blood flow quantification for arterial spin labeled perfusion MRI by removing residual motion artifacts and global signal fluctuations. Magn Reson Imaging.

[49] Goveas J, Xie C, Wu Z, Douglas WB, Li W, Franczak MB, Jones JL, Antuono PG, Yang Z, Li SJ (2011). Neural correlates of the interactive relationship between memory deficits and depressive symptoms in nondemented elderly: resting fMRI study. Behav Brain Res.

